# Endophytic *Pseudomonas koreensis* A1 of *Bletilla striata* as a Plant Growth Promoter and Biocontrol Agent Against Rice Sheath Blight

**DOI:** 10.3390/plants14223546

**Published:** 2025-11-20

**Authors:** Jian-Wei Jiang, Yue Qiu, Jing-Xue Luo, Jia-Le Liu, Hua-Jian Feng, Yi Zhou, Sheng Cheng

**Affiliations:** 1 College of Agriculture, Yangtze University, Jingzhou 434025, China; 2 School of Pharmacy, Xinyang Agriculture and Forestry University, Xinyang 464000, China

**Keywords:** *Pseudomonas koreensis*, *Rhizoctonia solani*, *Bletilla striata*, rice sheath blight, biocontrol

## Abstract

Rice sheath blight caused by *Rhizoctonia solani* is a devastating global rice disease. This study aimed to isolate biocontrol bacteria from the medicinal plant *Bletilla striata* for managing the disease. Strain A1 demonstrated the strongest antagonistic activity, with a 91.92% inhibition rate against *R. solani* in vitro. It also exhibited a broad antifungal spectrum against ten plant pathogenic fungi. Morphological and molecular (16S rRNA and *recA* genes) analysis identified strain A1 as *Pseudomonas koreensis*. In detached leaf assays, lesion length was significantly reduced. Pot and field trials showed control efficacies of 65.54% and 72.53%, respectively, comparable to the chemical agent Jinggangmycin. Strain A1 secreted extracellular enzymes (protease, β-1,3-glucanase), siderophores, and auxin (IAA), and possessed phosphate-solubilizing and nitrogen-fixing capabilities. The strain significantly enhanced the activities of key defense enzymes (POD, PAL, PPO, CAT, SOD) in rice. Furthermore, both its sterile culture filtrate and the corresponding crude ethyl acetate extract exhibited strong, direct suppression of *R. solani* growth. LC-MS analysis identified potential antifungal compounds, including Pseudomonic Acid, Artemisinin, and Tetradecane, in the extract. In conclusion, *P. koreensis* A1 is a promising biocontrol and plant growth-promoting candidate for sustainable management of rice sheath blight.

## 1. Introduction

As a primary staple food for more than half of the global population, rice (*Oryza sativa* L.) is a critically important cereal crop worldwide [1]. Rice sheath blight, caused by *Rhizoctonia solani*, is a devastating disease capable of infecting rice plants throughout their growth cycle, leading to yield losses exceeding 50% [2,3]. The severity of this disease necessitates effective control strategies to mitigate crop loss and prevent epidemics [4]. Currently, chemical control using Jinggangmycin is the primary method. However, prolonged use of chemical fungicides has led to reduced efficacy, pathogen resistance, and environmental pollution. Consequently, there is an urgent need for environmentally friendly alternatives to control *R. solani*. Biological control, being eco-friendly and less prone to inducing resistance, is increasingly recognized as the best future alternative to chemical pesticides.

To date, biological control of rice sheath blight has primarily focused on bacteria, especially endophytic bacteria [5]. For instance, *Bacillus* sp. [6], *Paenibacillus* sp. [2] and *Burkholderia* sp. [7] have been studied and proven effective for field control. Nevertheless, current biocontrol agents have not consistently performed well under field conditions. The discovery of novel, stable, and effective biocontrol strains therefore remains a critical priority.

*Pseudomonas* species are ubiquitous in various environments, including soil, water, plant rhizospheres, sediments, and even extreme habitats. They have simple nutritional requirements and play significant roles in plant health as rhizosphere or endophytic bacteria [8]. *Pseudomonas* protects plants from pathogens by producing diverse bioactive metabolites, such as siderophores, antibiotics, hydrolases, volatiles, and phytoanticipins, and through competition [9]. Additionally, they can enhance disease resistance by promoting plant growth and inducing defense-related enzymes [9,10]. For example, volatile organic compounds (VOCs) emitted by *P*. *fluorescens* ZX effectively suppress tomato gray mold [11]. The antimicrobial activity of *Pseudomonas* sp. B17-12 against tomato gray mold and fusarium wilt is likely due to volatile compounds like tetradecane [12]. *Pseudomonas fluorescens* strain VSMKU3054 significantly increases defense enzyme levels such as peroxidase (POX), phenylalanine ammonia-lyase (PAL), lipoxygenase (LOX), and total phenolic content in tomato plants, while moderately enhancing polyphenol oxidase (PPO) activity [9].

Endophytic bacteria are important sources of biocontrol agents. They function through various mechanisms, including antagonistic metabolite production, niche competition, plant growth promotion, and induced systemic resistance [13]. Studies indicate that many endophytic bacteria from medicinal plants possess antagonistic effects against plant pathogens [14]. For instance, Mohamad et al. [15] isolated over 100 endophytic bacteria from *Glycyrrhiza glabra*, several of which showed effective antifungal activity against eight different plant pathogenic fungi. *Bacillus amylolyticus* G146, isolated from *Fraxinus* spp, exhibited strong control against cotton verticillium wilt [16]. This suggests that medicinal plant endophytes provide valuable resources for developing novel biocontrol agents.

*Bletilla striata*, an Orchidaceae family medicinal plant primarily distributed in East Asia, exhibits pharmacological activities including wound healing, antimicrobial, anticancer, antioxidant, and antiviral properties [17,18]. The low disease prevalence in *B. striata* suggests it may host a unique endophytic microbiome with great biocontrol potential. Therefore, this study aimed to: isolate and identify endophytic bacteria from *B. striata* with high inhibitory effect against *R. solani*; evaluate the biocontrol effectiveness of *P. koreensis* A1 against rice sheath blight; and elucidate its potential mechanisms of action, including the production of antifungal compounds and induction of host defense responses.

## 2. Results

### 2.1. Isolation, Screening and Identification of Biocontrol strain A1

**Figure 1:**
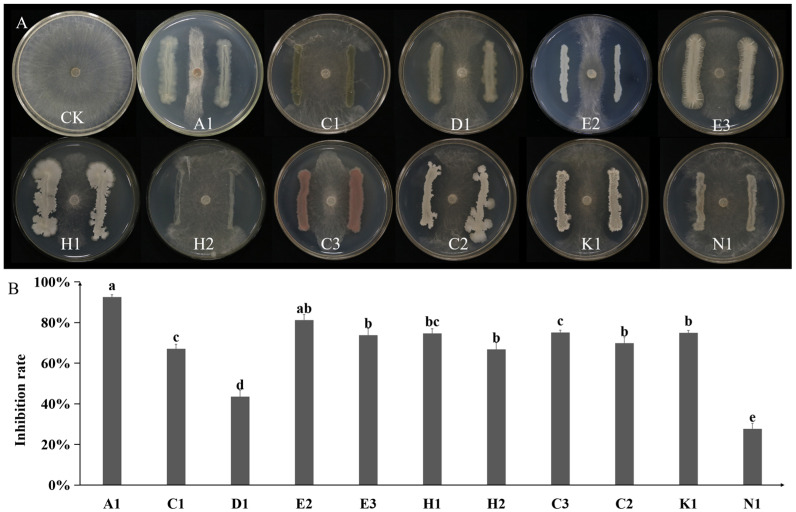
(**A**) Dual-culture assay of eleven selected endophytic bacterial strains of *Bletilla striata* against *Rhizoctonia solani*. (**B**) Inhibition rates of the eleven selected endophytic bacterial strains against *R. solani*. Data are presented as mean ± standard error, with different letters above bars denoting significant differences (*p* < 0.05, Duncan’s test).

**Figure 2:**
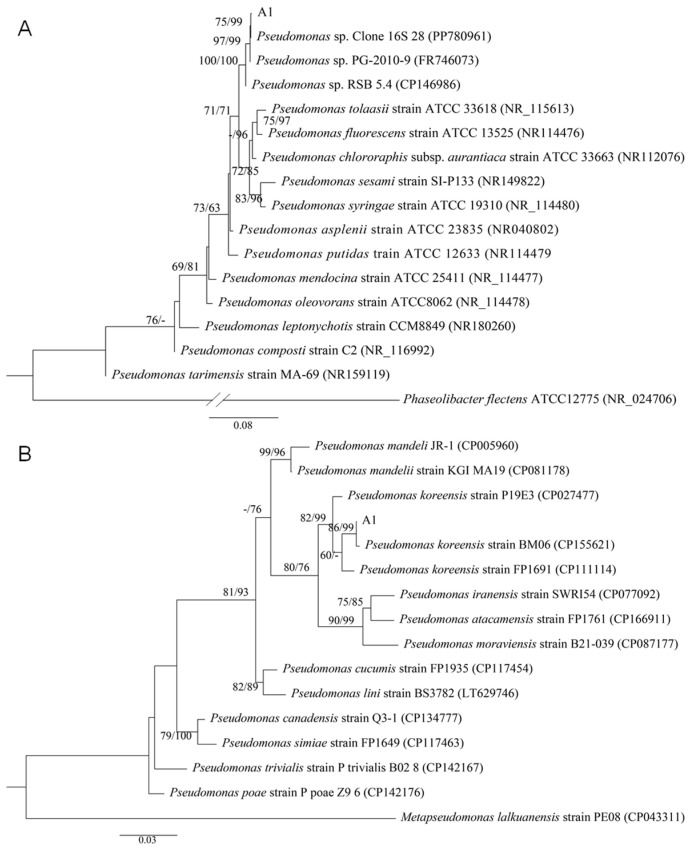
Phylogenetic analysis of strain A1, based on 16S rDNA (**A**) and *recA* (**B**) gene sequences, was conducted using the maximum likelihood (ML) and neighbor joining (NJ) method in MEGA 7.0. Bootstrap values of ML/NJ from 1000 replicates are shown at nodes. *Phaseolibacter flectens* ATCC 12,775 (NR_024706) and *Metapseudomonas lalkuanensis* strain PE08 (CP043311) were chosen as the outgroup.

Sixty-two endophytic bacterial strains were isolated from *Bletilla striata* roots. Eleven showed antagonistic activity against *Rhizoctonia solani* in dual-culture assays, with inhibition rates ranging from 42.42% to 92.28% (Figure 1A,B). Strain A1, with the highest inhibition rate (92.28%, Figure 1B), was selected for further study. Phylogenetic analysis based on 16S rDNA and *recA* gene sequences showed that strain A1 grouped within the *P. koreensis* cluster (Figure 2). Sequences were deposited in GenBank under accession numbers PV195001 (16S rDNA) and PV282236 (*recA*).

### 2.2. Antifungal Spectrum of Pseudomonas koreensis A1

**Figure 3:**
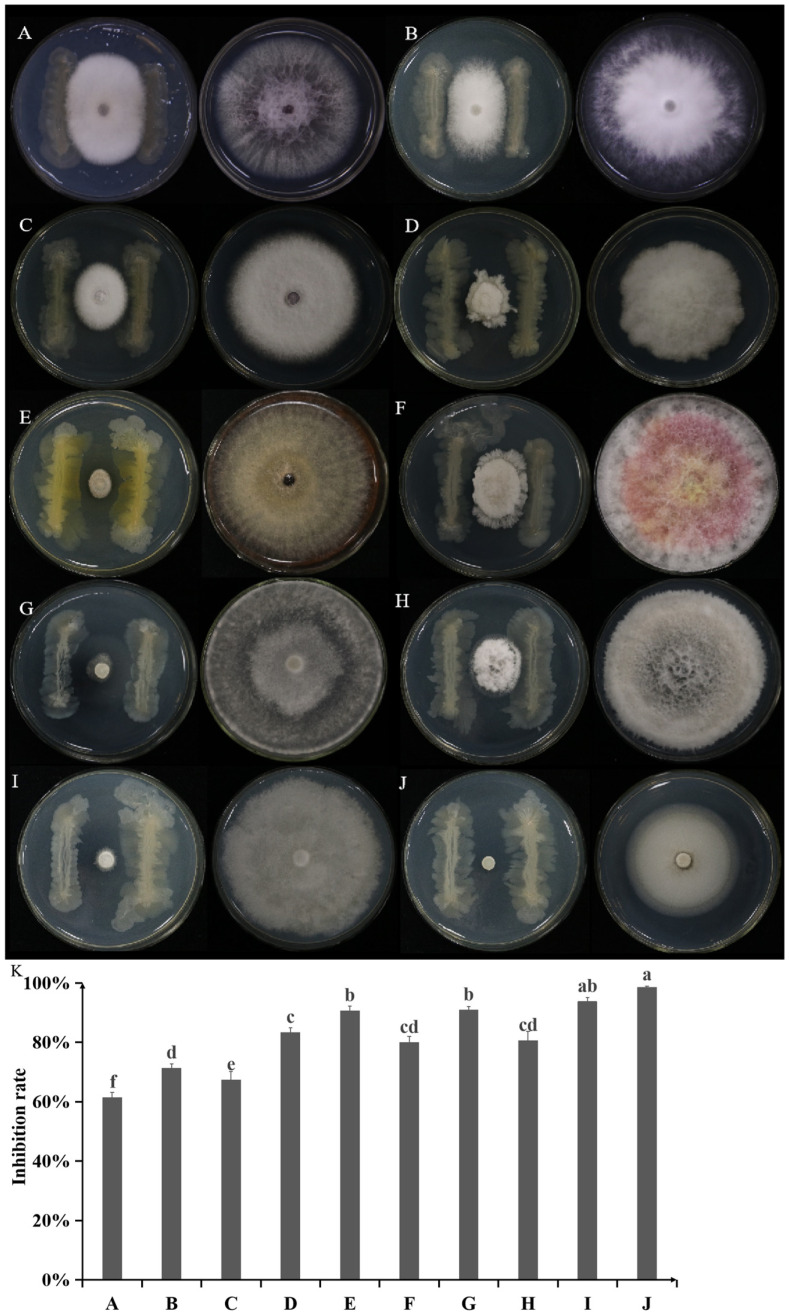
Antifungal spectrum of strain A1 against ten plant pathogenic fungi. Inhibition zones of *Bipolaris sorokiniana* (**A**), *Phytophthora infestans* (**B**), *Fusarium oxysporum* f. sp. *vasinfectum* (**C**), *F*. *fujikuroi* (**D**), *Alternaria solani* (**E**), *F*. *graminearum* (**F**), *Sclerotinia sclerotiorum* (**G**), *A. solani* (**H**), *P*. *nicotianae* (**I**), and *Verticillium dahlia* (**J**) are visible in dual-culture plates. (**K**) Bar chart shows corresponding inhibition rates. Data are presented as mean ± standard error, with different letters above bars denoting significant differences (*p* < 0.05, Duncan’s test).

Strain A1 displayed a broad-spectrum antifungal activity against all ten plant pathogenic fungi tested. The inhibition rates varied, with the highest observed against *Verticillium dahlia* (98.45%) and the lowest against *Bipolaris sorokiniana* (61.28%). The average inhibition rate for the other eight pathogens was 82.35%, demonstrating its potential as a broad-spectrum biocontrol agent (Figure 3).

### 2.3. Biocontrol Efficacy of P. koreensis A1 Against Rice Sheath Blight

**Figure 4:**
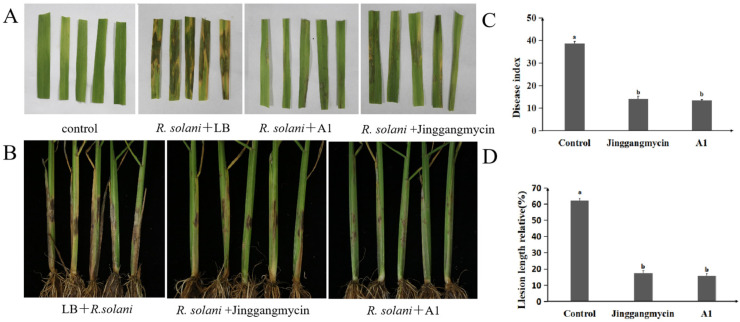
Biocontrol efficacy of *Pseudomonas koreensis* A1 against rice sheath blight in detached leaf and greenhouse assays. (**A**) Detached leaf bioassay: Lesion length on rice leaves treated with A1 fermentation broth, Jinggangmycin or LB medium after inoculation with *R. solani*. (**B**) Greenhouse pot assay: Disease index of rice plants treated with A1 fermentation broth, Jinggangmycin, or LB medium, following challenge with *R. solani*. (**C**) The Disease index of the rice leaves. (**D**) The Lesion length relative of the rice plants. Data are presented as mean ± standard error, with different letters above bars denoting significant differences (*p* < 0.05, Duncan’s test).

In the detached leaf assay, A1 fermentation broth significantly alleviated disease symptoms, reducing lesion area to 11.94%, comparable to that for the Jinggangmycin treatment (14.16%) and significantly lower than that for the LB control (62.06%) (Figure 4A,C). Strain A1 efficacy was further validated in greenhouse and field trials. In the greenhouse, A1 fermentation broth provided 82.90% control efficacy. Notably, under field conditions, A1 control efficacy reached 72.53%, statistically comparable to Jinggangmycin (69.63%) and significantly higher than the control (Table 1). These results confirm the strong potential of strain A1 for practical application.

### 2.4. Strain A1 Promotes Rice Seedling Growth

**Figure 5:**
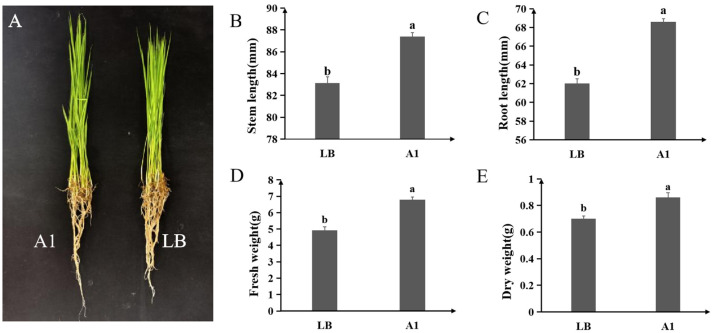
Plant growth-promoting effects of *P. koreensis* A1 on rice seedlings. (**A**) Phenotype of seedlings after treatment with A1 fermentation broth or LB medium. Stem length (**B**), root length (**C**), fresh weight (**D**), and dry weight (**E**) of rice seedlings. Data are presented as mean ± standard error, with different letters above bars denoting significant differences (*p* < 0.05, Duncan’s test).

Application of A1 fermentation broth significantly enhanced rice seedling growth under greenhouse conditions. Compared to the LB-treated control, A1-treated seedlings showed increases of 5.10% in stem length, 10.61% in root length, 38.55% in fresh weight, and 22.91% in dry weight (Figure 5A–E).

### 2.5. Antifungal Activity of Culture Filtrate and Crude Extract

**Figure 6:**
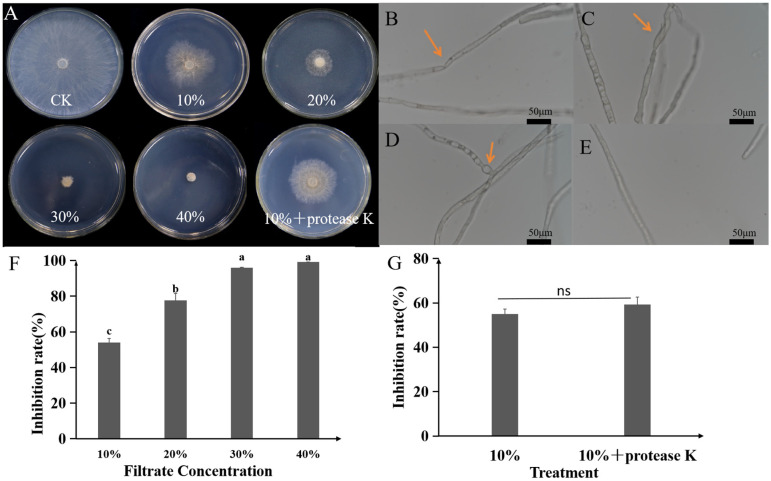
Inhibitory effect of the sterile culture filtrate (SCF) from *P. koreensis* A1 on the mycelial growth and morphology of *R. solani*. (**A**) Dose-dependent inhibition of *R. solani* by different concentrations of SCF after incubation at 28 °C for 2 days. (**B**–**D**) Microscopic observations (40× magnification) of abnormal hyphal morphology induced by SCF treatment, including shrinkage (**B**), deformation (**C**), and swelling (**D**). (**E**) Normal hyphal growth in the LB medium control. (**F**) Bar chart quantifying the inhibition rate of SCF at different concentrations. Data are shown as mean ± standard error. Data are presented as mean ± standard error, with different letters above bars denoting significant differences (*p* < 0.05, Duncan’s test). (**G**) Bar chart comparing the inhibition rate of 10% SCF with or without protease K treatment, showing that the antifungal activity was largely unaffected by protease. Data are presented as mean ± standard error. The absence of significant differences (*p* ≥ 0.05) between columns was indicated by “ns” according to Duncan’s test.

**Figure 7:**
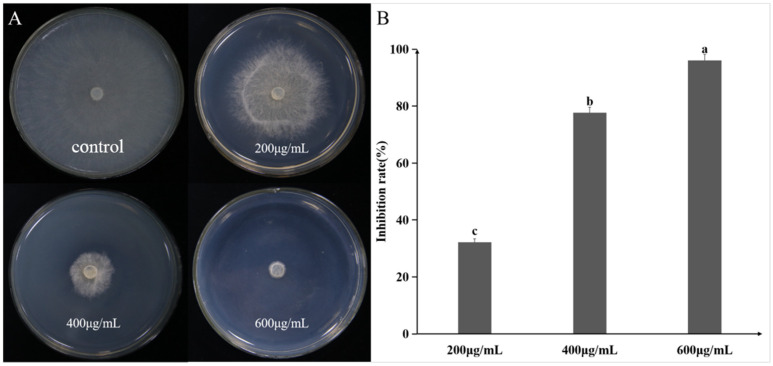
Antifungal activity of *P. koreensis* A1 crude lipopeptide extract against *R. solani*. (**A**) Dose-dependent inhibition of *R. solani* by different concentrations of crude lipopeptide extracts from *P. koreensis* A1 after incubation at 28 °C for 2 days. (**B**) Inhibition rate calculated based on radial growth compared to the control. Data are presented as mean ± standard error, with different letters above bars denoting significant differences (*p* < 0.05, Duncan’s test).

The sterile culture filtrate (SCF) of strain A1 exhibited dose-dependent inhibition against *R. solani* mycelial growth, with inhibitory activity progressively increasing at higher SCF concentrations (Figure 6A,F). Notably, 100% growth inhibition was achieved at 40% (*v*/*v*) SCF concentration (Figure 6A,F). Microscopic observation revealed that SCF treatment caused severe hyphal morphological abnormalities, including shrinkage, swelling, and distortion (Figure 6B–E). Treatment with protease K did not significantly reduce SCF antifungal activity (Figure 6G), suggesting the active components are not primarily proteinaceous. The ethyl acetate crude extract of A1 fermentation broth also exhibited potent dose-dependent antifungal activity against *R. solani*, achieving an inhibition rate of 96% at 600 μg/mL (Figure 7).

### 2.6. LC-MS Analysis of the Antifungal Crude Extract

LC-MS analysis of the crude extract identified several compounds with known antimicrobial properties, including Artemisinin, Pseudomonic Acid, Tetradecane, 2-Undecanone, and 2-Nonanone (Table 2 and Supplementary Figure S1). These compounds likely contribute to the direct antagonistic activity of strain A1.

### 2.7. Plant Growth-Promoting and Biochemical Traits of P. koreensis A1

**Figure 8:**
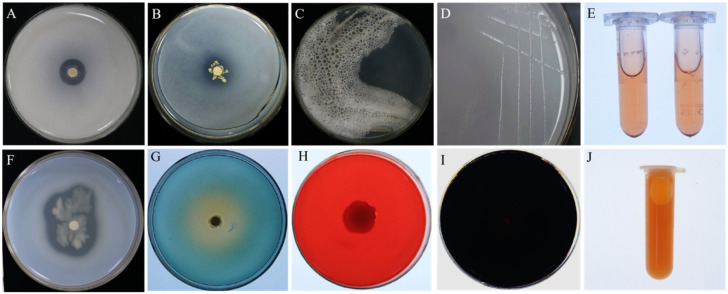
Functional characterization of *P. koreensis* A1 for plant growth-promoting and biocontrol traits. Qualitative assays confirm the strain’s ability to: solubilize potassium (**A**) and phosphate (**B**); produce catalase (**C**), IAA (**E**), siderophores (**G**), protease (**F**), and ammonia (**J**); and grow on a nitrogen-free medium (**D**), indicating nitrogen fixation ability. In contrast, activities for cellulase (**H**) and amylase (**I**) were not detected.

Strain A1 exhibited multiple plant growth-promoting traits. It produced siderophores, auxin (IAA) (Figure 8E), and ammonia (Figure 8J), and showed abilities to solubilize inorganic phosphate, release potassium, and fix nitrogen (Figure 8A,B,D). It also secreted several extracellular enzymes, including protease and β-1,3-glucanase, but not cellulase or amylase (Figure 8F–I). Catalase activity was confirmed (Figure 8C).

### 2.8. Strain A1 Primes Defense-Related Enzymes in Rice

**Figure 9:**
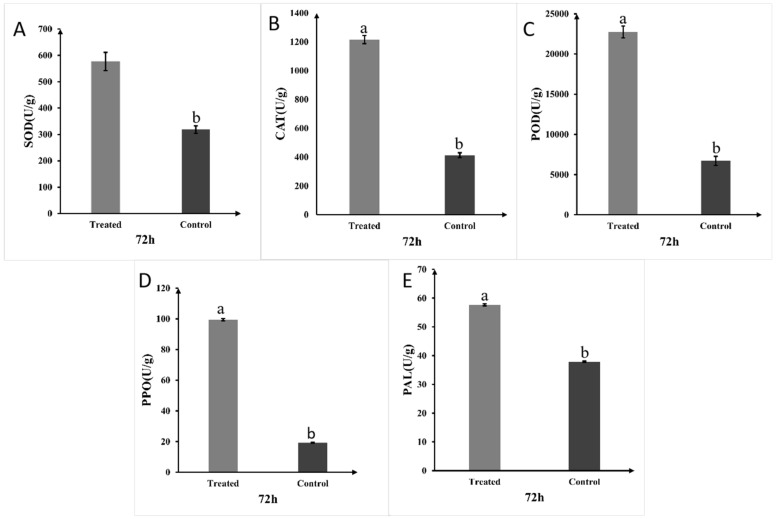
Activities of defense-related enzymes in rice seedlings 72 h after treatment with strain A1. The activities of SOD (**A**), CAT (**B**), POD (**C**), PPO (**D**), and PAL (**E**) were significantly increased in the A1-treated group compared to control (LB medium). Data are presented as mean ± standard error, with different letters above bars denoting significant differences (*p* < 0.05, Duncan’s test).

Application of A1 fermentation broth significantly induced systemic resistance in rice plants. At 72 h post-treatment, activities of key defense-related enzymes—polyphenol oxidase (PPO), catalase (CAT), phenylalanine ammonia-lyase (PAL), superoxide dismutase (SOD), and peroxidase (POD)—were significantly enhanced by 375%, 200%, 52.6%, 50%, and 275%, respectively, compared to the control (Figure 9).

## 3. Discussion

The search for potent biocontrol agents from unique ecological niches is a promising strategy for sustainable agriculture. Medicinal plants often possess antiparasitic, antibiotic, antioxidant, and anticancer activities [19]. Endophytes within these plants can synthesize bioactive secondary metabolites similar to their hosts, making medicinal plants valuable reservoirs for biological control agents [14,20,21,22]. This study isolated an endophytic bacterium, *Pseudomonas koreensis* A1, from the medicinal plant *Bletilla striata*, demonstrating remarkable potential for controlling rice sheath blight and promoting plant growth. Previous studies reported *P. koreensis* as a biocontrol agent against diseases caused by *Bipolaris sorokiniana*, *Fusarium solani* and *Alternaria alternata* [23,24,25]. To our knowledge, this is the first report of *P. koreensis* isolated from *B. striata* exhibiting inhibitory effects against rice sheath blight, expanding the host range and biocontrol potential of this species.

*Pseudomonas koreensis* A1 inhibited eight pathogenic fungi with inhibition rates exceeding 70%, indicating broad-spectrum potential. Furthermore, it showed high in vitro inhibition of *R. solani* and provided significant control in detached leaf, greenhouse, and field experiments, with efficacy comparable to Jinggangmycin. Thus, *P. koreensis* A1 demonstrates significant potential for development as a biopesticide. The slightly reduced field efficacy compared to the greenhouse is common, attributable to complex environmental factors like microbial competition, fluctuating weather, and soil variability [26]. Nevertheless, field efficacy exceeding 72% underscores its practical utility.

The biocontrol mechanism of *P. koreensis* A1 appears multifaceted. Our LC-MS analysis identified several antifungal compounds in the crude extract, including Pseudomonic Acid, Artemisinin, and volatile organic compounds such as Tetradecane and 2-Nonanone. Pseudomonic Acid (also known as Mupirocin), a clinically important antibiotic, exhibits broad-spectrum bactericidal activity against both Gram-positive (e.g., *Staphylococcus aureus*, including methicillin-resistant strains) and Gram-negative bacteria (e.g., *Escherichia coli*) [27]. Its primary mechanism involves the reversible inhibition of isoleucyl-tRNA synthetase, which disrupts bacterial protein synthesis [28]. While its role in antifungal activity is less defined, it is plausible that the compound targets a similar pathway in fungi or exerts its effect through alternative mechanisms, such as disrupting fungal membrane integrity. Artemisinin exhibits antifungal efficacy against a range of fungi, including *Aspergillus* niger, *Fusarium* spp., *Trichoderma* spp. and *A. flavus* [29,30]. A key mechanism of this growth inhibition is the induction of oxidative stress via reactive oxygen species generation [31]. Meanwhile, volatile compounds like Tetradecane and 2-Nonanone are frequently associated with membrane permeability alterations and inhibition of hyphal growth and sporulation in fungi. Tetradecane synergistically enhances antifungal activity against *Candida albicans* [32,33]. 2-Undecanone and 2-Nonanone combat fungi by inhibiting biofilm formation, blocking yeast-hypha transition, and interacting with Hwp1 protein [34,35]. The diverse arsenal of bioactive molecules produced by A1 enables a concerted action against *R. solani*, simultaneously compromising its cellular integrity, metabolic processes, and redox homeostasis. This multi-targeted attack results in a synergistic reduction in fungal pathogenicity and invasiveness, underpinning the strain’s broad-spectrum antifungal activity.

Biocontrol bacteria can produce extracellular enzymes to suppress pathogen growth [36,37,38]. In addition to direct antifungal effects, *P*. *koreensis* A1possesses an array of plant growth-promoting traits, including secretion of extracellular enzymes, siderophores, IAA, and ammonia, and abilities to solubilize phosphate and potassium and fix nitrogen. These traits directly translated into enhanced rice seedling growth, establishing *P. koreensis* A1 as a plant growth-promoting rhizobacterium (PGPR). Healthier plants are inherently more disease-resistant.

Moreover, *P. koreensis* A1 acts as an elicitor of plant innate immunity. We demonstrated significant and simultaneous upregulation of five key defense enzymes (POD, PAL, PPO, CAT, SOD) in A1-treated rice plants. By scavenging intracellular reactive oxygen species (ROS), these enzymes bolster plant immunity, thereby activating defense signaling pathways dependent on salicylic acid (SA), jasmonic acid (JA), and ethylene (ET) [39,40]. This “priming” of defense responses prepares the plant for a more robust and rapid defense upon pathogen challenge [41,42]. The coordinated increase in these enzymes, involved in lignin synthesis, phenolic compound production, and ROS scavenging, creates a hostile environment for invading pathogens like *R. solani* [43,44]. In conclusion, the synergistic combination of multiple antagonistic and plant growth-promoting mechanisms makes *P. koreensis* A1 a robust and promising multi-functional bio-agent.

It is important to acknowledge a key limitation of this study. While our in vitro and detached leaf assays provide compelling evidence for the direct antifungal activity of A1 and its metabolites, the experimental design does not allow us to definitively dissect the relative contributions of direct inhibition versus induced host immunity in the biocontrol efficacy observed in plant. In planta, it is highly plausible that both mechanisms operate synergistically. The direct antagonism reduces the pathogen load, while the simultaneously induced systemic resistance (as evidenced by the primed defense enzymes) enhances the plant’s innate ability to confine the infection. Disentangling this complex interplay, for example, through gene knockout mutants of specific bacterial traits or the use of plant signaling mutants, represents a fascinating and necessary direction for future research to fully unravel the mechanistic basis of this promising biocontrol agent.

## 4. Materials and Methods

### 4.1. Isolation of Endophytic Bacteria from Bletilla striata Roots

Healthy *B. striata* specimens were collected from the Yangtze University Industrial Park, Jingzhou City, Hubei province. Roots were thoroughly washed with running water and externally sterilized by sequential submersion in 75% ethanol (2 min), 4% sodium hypochlorite (3 min), and 75% ethanol (1 min), followed by five rinses with aseptic distilled water. The effectiveness of surface disinfection was verified by plating the last rinse water on Luria–Bertani agar (LA). Sterilized root tissues were aseptically ground with sterile quartz sand and serially diluted. Aliquots (100 μL) of dilutions (10^4^ to 10^6^) were spread on LA plates and incubated at 28 °C for 3 days. Morphologically distinct colonies were purified and subsequently preserved at −80 °C in 15% glycerol for long-term storage.

### 4.2. Evaluation of Antifungal Activity Against Rhizoctonia solani

Antagonistic activity was evaluated using a dual-culture assay. A 6-mm-diameter mycelial plug from a 3-day-old *R*. *solani* culture was placed at the center of a fresh potato dextrose agar (PDA) plate. Eleven bacterial isolates from purified plates were streaked as two parallel lines at a distance of 20 mm from the fungus. Plates inoculated with *R. solani* alone served as negative control group. All plates were incubated at 28 °C for 2 days, after which radial fungal growth was measured. The inhibition rate was calculated as: [(Rc − Rt)/Rc] × 100, where Rc is radial growth in the control, and Rt is radial growth in the treatment.

### 4.3. Molecular Identification of Strain A1

Under culture conditions of LB medium, 28 °C, and shaking at 130 rpm, strain A1 was grown overnight. Genomic DNA was extracted using a bacterial genome extraction kit (Tsingke Biotechnology Co., Ltd., Wuhan, China). The 16S rRNA and *recA* genes were amplified with primer pairs 27F/1492R and recA-F/recA-R [45]. Purified PCR products were sequenced by Tsingke Biotechnology Co., Ltd. (Wuhan, China). Obtained sequences were analyzed using the NCBI BLAST web server (https://blast.ncbi.nlm.nih.gov/Blast.cgi, accessed on 20 October 2025) against the GenBank database. The maximum likelihood (ML) and neighbor joining (NJ) method in MEGA 7.0 was used to infer phylogenetic trees, assessing node confidence with 1000 bootstrap replications [44].

### 4.4. Assessment of Antifungal Spectrum

Strain A1, exhibiting the strongest antibacterial activity against *R. solani*, was selected for the antifungal spectrum test against ten plant pathogenic fungi: *Bipolaris sorokiniana*, *Phytophthora infestans*, *Fusarium oxysporum* f. sp. *vasinfectum*, *F*. *fujikuroi*, *Alternaria solani*, *F*. *graminearum*, *Sclerotinia sclerotiorum*, *Verticillium dahlia*, *A. solani* and *P*. *nicotianae* as preserved in the Fungi Herbarium of Yangtze University (Jingzhou, China). The dual-culture assay was performed as described in Section 4.2, and inhibition rates were calculated.

### 4.5. Antifungal Activity of Sterile Culture Filtrate

Strain A1 was grown in LB medium for 7 days at 28 °C with shaking at 200 rpm. The culture was centrifuged and filter-sterilized (0.22 μm pore size) to obtain the sterile culture filtrate (SCF). The SCF was incorporated into PDA at concentrations of 10%, 20%, 30%, and 40% (*v*/*v*) [46]. To test sensitivity to proteolysis, the SCF was treated with protease K (100 μg/mL) at 28 °C for 1 h before being added to PDA at 10% (*v*/*v*). A 6-mm mycelial plug of *R. solani* was positioned centrally on each plate, and mycelial growth was measured after incubation at 28 °C. The inhibition rate was assessed following the protocol in Section 4.2.

### 4.6. Detached Leaf Assay

Healthy, five-week-old rice leaves were cut into 5 cm segments and surface-disinfected with 2.5% (*v*/*v*) sodium hypochlorite for 30 s, subsequently rinsed three times with sterile water. Leaf segments were divided into three treatment groups and immersed for 1 h in 100 mL of either: A1 fermentation broth (OD_600_ = 1.0), Jinggangmycin (0.5 mg/mL), or LB medium. Each leaf segment was then inoculated at the center with a 6-mm mycelial plug obtained from a 2-day-old colony *R. solani* culture. Treated leaves were maintained at 28 °C with high humidity for 5 days, after which lesion length was measured.

### 4.7. Greenhouse Assessment of Biocontrol Efficacy

Seeds of rice cultivar ‘Xudao No. 9’ were surface-disinfected and germinated on sterile moist filter paper at 28 °C. Uniformly germinated seeds were transplanted into plastic pots containing sterilized soil and grown in a greenhouse at 28 °C, 90% relative humidity, and with a photoperiod of 16 h light and 8 h darkness. Upon reaching the 4–5 leaf stage, plants were grouped into three treatments: (1) *R. solani* + A1 fermentation broth (1.0 × 10^8^ CFU/mL), (2) *R. solani* + Jinggangmycin (0.5 mg/mL), and (3) *R. solani* + LB medium. Treatments were applied as foliar sprays twice at a one-week interval. Twenty-four hours after the first spray, *R. solani* sclerotia were attached to leaf sheaths near the plant base using sterile insect needles. Plants were enclosed transparent plastic bags for 48 h to sustain elevated humidity levels. Disease severity was assessed 14 days post-inoculation using a 0–9 scale standard (IRRI, 1980). The disease index and relative control efficacy were calculated as follows:

Disease index = [∑(Number of diseased plants in a grade × Disease grade)/(Total number of plants × Highest disease grade)] × 100.

Control efficacy (%) = [(Disease index of control − Disease index of treated group)/Disease index of control] × 100.

### 4.8. Field Trial for Control of Rice Sheath Blight

A field trial was conducted in Jingzhou, Hubei Province, in a site with a history of severe rice sheath blight. Rice cultivation followed local conventional practices. The area experiences frequent and severe natural sheath blight occurrence, with recent incidence rates reaching 70% and disease indices between 30 and 40. The experimental field was divided into 12 plots (30 m^2^ each) using a randomized block design, with 45 cm spacing between plots. Two treatments were applied: A1 fermentation broth (1 × 10^8^ CFU/mL) and LB medium. At the early onset of sheath blight, treatments were applied via foliar spray. Disease severity was assessed 10 days post-treatment by randomly selecting 20 plants per plot. Disease evaluation and control efficacy calculations followed Section 4.7.

### 4.9. Antifungal Activity of Crude Extract

Antifungal active compounds were extracted following Mandal et al. [47]. A1 was inoculated into 100 mL LB broth and cultivated at 28 °C with shaking at 150 rpm for 24 h. Then, 10 mL of the fermented broth was transferred into 1 L LB medium and cultivated under the same conditions for one week. To obtain the supernatant, the fermented broth was subjected to centrifugation at 10,000 rpm for 20 min. The supernatant was combined with an equal volume of ethyl acetate, vigorously shaken in a separatory funnel, and then allowed to stand for phase separation. Following three rounds of extraction, the combined organic phases were concentrated using a rotary evaporator at 35 °C. The dried extract was weighed and dissolved in mass spectrometry-grade methanol to 20 mg/mL. The preparation was filtered through a 0.22 µm filter for subsequent experiments. The crude extract was incorporated into PDA medium to achieve final concentrations of 50, 100, 200, and 500 µg/mL. A mycelial plug of *R. solani* was aseptically transferred to the center of each PDA plate, with three replicates per concentration, and after incubation at 28 °C for two days, the mycelial diameter was measured for the subsequent determination of the inhibition rate.

### 4.10. LC-MS Analysis of Antifungal Metabolites

Components of the lipopeptide active substances were analyzed using high-resolution liquid chromatography-mass spectrometry (LC-MS). The crude extract from Section 4.9 was diluted to 5 mg/mL with methanol, sterilized by filtration through a 0.22-μm membrane into dedicated sample vials. Chromatography employed a Kinetex^®^ F5 column (100 mm × 2.1 mm, 2.6 μm), with ultrapure water as mobile phase A and LC-MS grade acetonitrile as mobile phase B. Gradient elution was: 0–2 min, 5% B; 2–8 min, 5–60% B; 8–20 min, 60–95% B; 20–25 min, 95% B; 25–25.01 min, 95–5% B; and 25.01–27 min, 5% B. The injection volume was 10 μL, flow rate 300 μL/min, and detection wavelength 254 nm. Mass spectrometry used an ESI ion source in positive and negative ion modes. Ion spray voltage was 5000 V, ion source temperature 500 °C, column temperature 40 °C, and mass scanning range (*m*/*z*) 100 to 1250. Initial LC-MS data were interpreted using SCIEX OS 1.7.0 software. By comparing data with existing database information, the main components of the lipopeptide active substances were identified.

### 4.11. Characterization of Plant Growth-Promoting Properties

#### 4.11.1. Biosynthesis of Siderophores

Siderophore production was determined using the Chrome Azurol S (CAS) agar plate assay [48]. A 6 mm sterile filter disc was positioned at the center of the CAS plate, and 10 μL of strain A1 inoculum was inoculated onto it. Formation of orange halos indicated siderophore production.

#### 4.11.2. Phosphorus Solubilization

Phosphate solubilization ability was assessed using NBRIP agar medium [49]. Aseptic technique was used to place a 6-mm filter paper disc in the center of the NBRIP plate, inoculated with 10 μL of strain A1 suspension, and incubated in the dark at 28 °C for 7 days. The appearance of a clear zone demonstrated phosphate solubilization.

#### 4.11.3. Nitrogen Fixation

Nitrogen-fixing capacity was evaluated by streaking a fresh A1 colony from an LA plate onto a nitrogen-free medium [50] and incubating at 25 °C for 7 days. Colony growth was observed post-incubation.

#### 4.11.4. Extracellular Enzyme Activity

Activities of extracellular enzymes (protease, chitinase, cellulase) were determined using skim milk agar medium [51], carboxymethyl cellulose agar (CMC) [52], and colloidal chitin solid medium [53], respectively. The A1 bacterial suspension was inoculated onto these media. After incubation at 28 °C for 7 days, clear zones around colonies indicated positive enzymatic reactions.

#### 4.11.5. Indole-3-Acetic Acid (IAA) Production

Auxin production was assessed using the Salkowski method [50]. Strain A1 was cultured in LB medium with 10 g/L tryptophan at 28 °C and 200 rpm for 2 days. After centrifugation (10,000 rpm, 10 min), 2 mL supernatant was mixed with 2 mL Salkowski reagent (0.5 M FeCl_3_ in 35% HClO_4_). The mixture was maintained in darkness for 30 min. A color change to pink indicated IAA production.

### 4.12. Measurement of Defense-Related Enzyme Activities

Rice plants at the tillering stage received four treatments: A1 fermentation broth alone; LB medium. Leaf samples were collected 72 h after the final treatment. Activities of POD, PAL, PPO, CAT, and SOD were measured using commercial assay kits (Solarbio, Beijing, China) according to the manufacturer’s instructions.

### 4.13. Statistical Analysis

Statistical analyses were performed in SPSS 23.0 (IBM, Armonk, NY, USA). We conducted one-way ANOVA to evaluate overall variability across treatments. Where ANOVA indicated significance (*p* < 0.05), Duncan’s test was employed for pairwise comparisons, as it offers high detection power for experiments with multiple groups. Data are presented as mean ± standard error.

## 5. Conclusions

This study isolated an endophytic bacterial strain A1 with high antagonistic activity against *Rhizoctonia solani* from *Bletilla striat*, identified as *Pseudomonas koreensis*. The strain exhibited broad-spectrum antifungal activity, significant control efficacy against rice sheath blight in pot and field trials, and pronounced growth-promoting effects on rice. Its biocontrol mechanisms involve production of antifungal metabolites, multiple plant growth-promoting traits, and induction of host defense responses. Therefore, *P. koreensis* A1 represents a highly promising multi-functional biocontrol agent for the sustainable management of rice sheath blight.

## Figures and Tables

**Table 1:** Biocontrol activity of strain A1 fermentation broth and Jinggangmycin against rice sheath blight in field trials.

Treatment	Disease Index	Control (%)
Control (LB)	12.72 ± 0.95 a	-
Jinggangmycin	3.58 ± 1.13 b	69.63%
A1 (10^8^ cfu/mL)	2.94 ± 0.52 b	72.53%

Data are presented as mean ± standard error, with different letters above bars denoting significant differences (*p* < 0.05, Duncan’s test).

**Table 2:** Identification of antimicrobial compounds in the crude extract of *P. koreensis* A1 by LC-MS analysis.

Material Type	Peak Time	Mass/Charge
Artemisinin	6.035–6.071	281.1394
Pseudomonic Acid	7.278–7.314	499.2913
Tetradecane	7.314–7.349	197.2275
2-Undecanone	7.830–7.866	169.1598
2-Nonanone	8.046–8.082	141.1285

## Data Availability

Data are contained within the article and Supplementary Materials.

## Supplementary Materials

The following supporting information can be downloaded at: https://www.mdpi.com/article/10.3390/plants14223546/s1, Figure S1: LC-MS profile of antimicrobial compounds from *P. koreensis* A1. The analysis identified several antifungal metabolites in the crude extract, including Pseudomonic Acid (A), Artemisinin (B), 2-Nonanone (C), 2-Undecanone (D), and Tetradecane (E).
