# Supplementary material for: Endophytic Pseudomonas koreensis A1 of Bletilla striata as a Plant Growth Promoter and Biocontrol Agent Against Rice Sheath Blight
Source: Plants (Basel). 2025 Nov 20;14(22):3546. doi: 10.3390/plants14223546 (PMC12656357; doi:10.3390/plants14223546)
Supplement: Supplementary file 1 [file plants-14-03546-s001.zip › plants-3969756-supplementary.pdf]

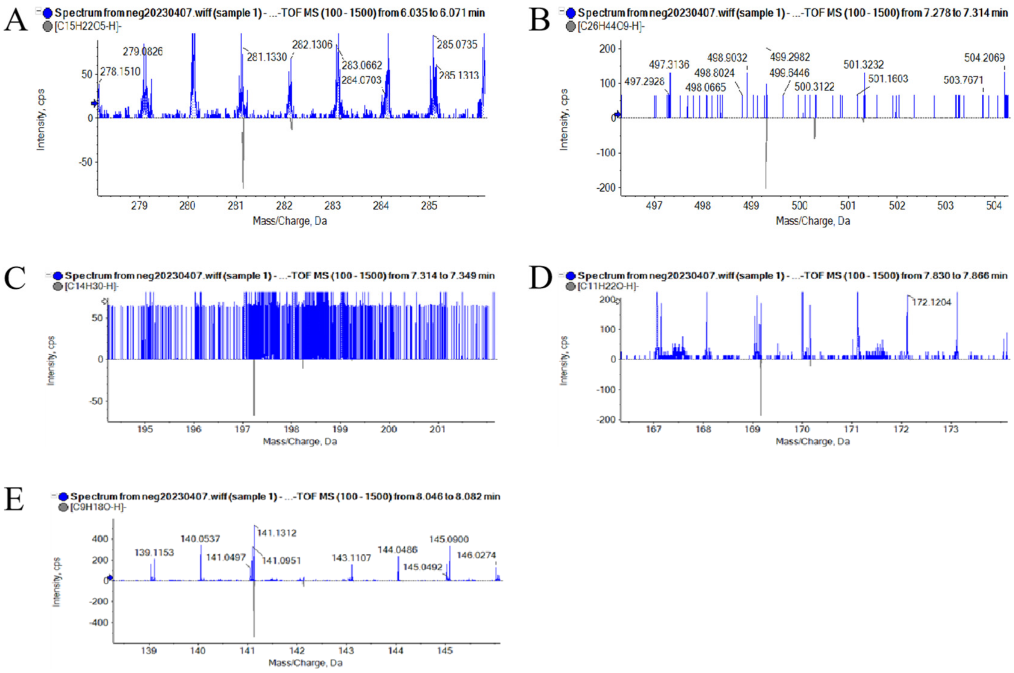

Figure S1. LC-MS profile of antimicrobial compounds from *P. koreensis* A1. The analysis identified several antifungal metabolites in the crude extract, including Pseudomonic Acid (A), Artemisinin (B), 2-Nonanone (C), 2-Undecanone (D), and Tetradecane (E).
